# Comparison of the Phytochemical Composition and Antibacterial Activities of the Various Extracts from Leaves and Twigs of *Illicium verum*

**DOI:** 10.3390/molecules26133909

**Published:** 2021-06-26

**Authors:** Emirlyn-Cheng Yang, Ya-Yun Hsieh, Li-Yeh Chuang

**Affiliations:** 1 Department of Biochemistry, The University of Hong Kong, Pokfulam, Hong Kong, China; emirlyny@connect.hku.hk; 2 Institute of Biotechnology and Chemical Engineering, I-Shou University, Kaohsiung 840, Taiwan; stephy1989106@gmail.com; 3 Department of Chemical Engineering, I-Shou University, Kaohsiung 840, Taiwan

**Keywords:** *Illicium verum*, supercritical fluid extraction, traditional solvent extraction, antimicrobial activity

## Abstract

Previous studies have revealed the numerous biological activities of the fruits of *Illicium verum*; however, the activities of its leaves and twigs have remained undiscovered. The study aimed to investigate the phytochemical components and antibacterial activity of the various extracts from the leaves and twigs of *Illicium verum*. The herbal extracts were prepared by supercritical CO_2_ extraction (SFE) and 95% ethanol extraction, followed by partition extraction based on solvent polarity. Analysis of antimicrobial activity was conducted through the usage of nine clinical antibiotic- resistant isolates, including *Staphylococcus aureus*, *Pseudomonas aeruginosa* and *Acinetobacter baumannii*. Among the tested samples, the SFE extracts exhibited broader and stronger antibacterial activities against the test strains, with a range of MIC between 0.1–4.0 mg/mL and MBC between 0.2–4.5 mg/mL. Observations made through scanning electron microscopy revealed potential mechanism of the antimicrobial activities involved disruption of membrane integrity of the test pathogens. Evaluation of the chemical composition by gas chromatography-mass spectrometry indicated the presence of anethole, anisyl aldehyde, anisyl acetone and anisyl alcohol within the SFE extracts, demonstrating significant correlations with the antibacterial activities observed. Therefore, the leaves and twigs of *Illicium verum* hold great potential in being developed as new natural antibacterial agents.

## 1. Introduction

For decade, due to the rapid development of medical technology, many antibiotics have been developed to increase human life expectancy. However, long-term abuse of antibiotics has led to mutations in several bacterial strains. There has been a ubiquitous emergence and dissemination of medically important multidrug-resistant microbial strains throughout the world, raising public health concerns in many countries, including those that are developed, developing, and underdeveloped [1,2]. *Staphylococcus aureus* (Sa), *Pseudomonas aeruginosa* (Pa) and *Acinetobacter baumannii* (Ab) are clinically important pathogens that have contributed significantly to worldwide nosocomial infections [3]. These bacterial strains were reported to possess high levels of resistance towards many classes of antibiotics. In addition, the bacterial strains have been listed as the top priority pathogens for the development of new antimicrobials by the World Health Organization (WHO) [4]. To prevent the growth of multiple antibiotic resistant and pan-drug-resistant bacteria, new antibiotics must be developed. In multiple countries, *Staphylococcus aureus*, *Pseudomonas aeruginosa* and *Acinetobacter baumannii* are the bacterial strains that exhibit the strongest antibiotic resistance [5]. 

For thousands of years, Chinese medicinal herbs have been studied extensively to improve health or cure diseases. In recent years, many literatures have indicated that there are many bioactivities found within Chinese medicinal herbs [6]. Thus, in this study, we investigated the antibacterial properties of Chinese medicinal herbs to develop natural antibiotics to substitute one that are chemically synthesized. 

In recent years, an increasing number of applications of supercritical fluid extraction (SFE) has been developed in the food industry. Due to the fact that CO_2_ is non-toxic, odorless, less expensive, environmental friendly and recyclable, it has been commonly used as the solvent in SFE. Furthermore, SFE not only enables extraction of effective substances through a variation of temperature or pressure but also preserves bioactive compounds of products effectively during low temperature extraction [7]. 

Traditional solvent extraction (TSE) is still commonly used in the food industry, with the use of solvents such as hexane, ethyl acetate, ethyl ether or alcohols. Among alcohols, ethanol possesses the advantages of being easy-to-use, cheap and highly efficient. Though methanol exhibits similar characteristics to ethanol, it can exert potent toxic effects in humans. Studies have revealed that humans are susceptible to loss of sight or even death upon misuse of methanol. In addition, solvent waste pollution and residues have been considered to be one of the greatest problems with using TSE. Thus, in consideration of the safety issues involved, ethanol was selected as the solvent [8].

During the last 50 years, many studies have indicated that antimicrobial agents derived from natural products exhibit reduced side effects, which underscores their potential to serve as a superior alternative for chemically synthesized drugs in therapy [9]. *Illicium verum*, also known as star anise or anise, is a small native evergreen tree found in northeast Vietnam and southwest China. In recent years, the bioactivities of *I. verum* fruits are widely investigated as many studies have revealed that it possesses many biological functions, including but not limited to antimicrobial, antioxidant, insecticidal, analgesic, sedative, anti-flu and convulsive activities [10,11,12,13]. Numerous studies have revealed that the essential oil of *I. verum* fruits exhibited high bioactivity levels, including antioxidant activity [14,15], insecticidal activity [16], antimicrobial activity [17,18] and antifungal activity [12,19]. In particular, anethole, the main component of *I. verum* oil, has been reported to play a critical role in the listed bioactivities [20,21,22].

Multiple studies have revealed that significant antimicrobial activities for different types of pathogens, including Gram positive bacteria, Gram negative bacteria, and numerous fungi, were observed from the application of essential oil and extracts from fruits of *I. verum*. [23,24,25,26]. In our previous study, we found that the ethyl ether fraction and supercritical CO_2_ extract of *I. verum* fruits exhibited antibacterial activity with MIC values of 0.15–0.70 mg/mL. Furthermore, four main antibacterial substances (*trans*-anethole, anisyl aldehyde, anisyl acetone and anisyl alcohol) from the active extracts were identified by GC-MS [27]. Ebani et al. (2018) investigated the antimicrobial activity of essential oils from *I. verum* against multiple resistant strains of various pathogens isolated from dogs and cats. Their results indicated that the essential oils from *I. verum* exhibited antibacterial activity against most of the test strains, with the exception of Enterococci [17]. In 2017, Rahman et al. reported that the *I. verum* extracts may serve as potential quorum sensing and biofilm inhibitors [28]. More recently, Salem et al. demonstrated that the *I. verum* aqueous methanol (50%) extract possesses antibacterial activity against multidrug resistant *Acinetobacter baumannii* and methicillin resistant *Staphylococcus aureus* based on the in vitro and in vivo analysis [11]. According to the complete list of polar metabolome they provided, more than 70 compounds from different classes were identified, including phenolics, phenylpropanoids, sesquiterpenes, tannins, lignans and flavonoids. In 2021, Li et al. developed a multistage extraction method to extract and separate various bioactive compounds from *I. verum*. Their work revealed that the various bioactive-rich extracts may be achieved by using steam distilled- or oxidative- extraction methods, which leads to high extraction yields with high purity. Moreover, their study also indicated that the extracts display antibacterial activities against various types of microorganisms, with the lowest MIC value being 400 μg/mL [29]. Although antimicrobial activities of *I. verum* fruits have been closely examined, previous studies have not determined the antibacterial effects and compositions of the leaves and twigs from *I. verum*.

Numerous compounds such as volatiles, seco-prezizaane-type sesquiterpenes, phenylpropanoids, lignans, flavonoids and other constituents have been identified from *I. verum* [26]. However, little progress has been made in terms of using supercritical CO_2_ extractions to extract bioactive compounds from the leaves and twigs of *I. verum*. In regard to the extracts of leaves and twigs, analysis of the antimicrobial activities against clinical antibiotic resistant strains has not been carried out. According to our previous study [27], we found that although the extracts of supercritical CO_2_ extracts from *I. verum* fruits showed lower antioxidant activity than the ethanol extracts, their antibacterial effects were more significant against *Acinetobacter baumannii.* Therefore, in this study, the supercritical CO_2_ fluid and ethanol extractions were used to extract the leaves and twigs of *I. verum*, followed by comparisons on their antibacterial activities and active compounds.

## 2. Results and Discussion

### 2.1. Extraction Yields of the Extracts from Leaves and Twigs of Illicium verum

The extraction yields of the supercritical CO_2_ extraction (SFE) and traditional solvent extraction (TSE) are shown in Table 1. In regard to the SFE results, the extraction yields of twigs (0.09–0.96%) were higher than that of leaves (0.01–0.59%). The extracts collected from the first 30 min (extract A) displayed the highest extraction yield among all the sample extracts. For results from TSE, the extraction yield of twigs (16.1%) were higher than leaves (14.27%). Similarly, results found in the partition fractions displayed extraction yields of 0.62–3.37% and 0.41–2.87%, respectively. Among the partition fractions, the hexane fractions obtained from both leaves (2.87%) and twigs (3.37%) exhibited the highest extraction yields.

The results indicated that the extraction yields of TSE were higher than that of SFE, which aligned with our expectations. Traditional solvent extraction was commonly used for natural product extraction. However, the use of TSE has been reported to present multiple disadvantages including influences on the environment, long time consumption, numerous unit operations, high energy consumption, requirement of large quantity of solvent, yield of poor quality extract and generation of more waste etc. Due to the disadvantages of TSE, the idea of green extraction has attracted attention in recent year. In 2019, Chemat et al. proposed the idea of green extraction, which focused on utilization of extraction technology that emanates the lowest influences on the environment while ensuring production of high quality products [30]. Thus, an environmentally friendly method for natural product extraction, supercritical CO_2_ extraction, was conducted for comparisons with the traditional methods in this study.

### 2.2. Antibacterial Activity of the Extracts from Leaves and Twigs of Illicium verum

#### 2.2.1. Disk Diffusion Assay

Preliminary tests to measure antimicrobial activity against nine clinical antibiotic resistant isolates (Sa2875, Sa2803, Sa0752, Pa4016, Pa4285, Pa3647, Ab3327, Ab2375, Ab3394) were conducted on the various sample extracts obtained from leaves and twigs of *Illicium verum.* The same strains differ in their phenotypic resistance against antibiotics, which may vary in the degree of resistance and type of antibiotics. As shown in Table 2, the SFE extracts of leaves and twigs of *I. verum* exhibited a better and broader spectrum of antibacterial abilities against all the test strains, with a DIZ range between 9–22 mm. In particular, SFE extract A showed significant antibacterial activities against Ab3327, Pa4016 and Sa2875 with DIZ of 22 mm, 16.5 mm and 11 mm, respectively. SFE extract B revealed significant antibacterial activity against Ab3327, Pa4285 and Sa2875 with DIZ of 20.5 mm, 17 mm and 16.5 mm, respectively. Extracts C, C_Hex_ and C_EA_ of SFE demonstrated mild antibacterial activities and distinct antibacterial effects toward different strains. For the TSE extracts comparison, the Hex and EA fractions of leaves and twigs of *I. verum* had a broader antibacterial activity and significant antibacterial effects against all the test isolates (Table 2). 

However, the leaves and twigs of EA fraction revealed more significant antibacterial activity than the leaves and twigs of Hex fraction with DIZ ranges of 10.0–16.0 mm and 9.0–22.5 mm, respectively. The ethanol crude extract did not demonstrate antibacterial activity against the Pa3647 strain. The aqueous fractions from both leaves and twigs exhibited the least antibacterial activity against the test strains. Through comparisons of results from the extracts obtained from FSE and TSE, it indicated that the FSE extracts B from both leaves and twigs exhibited higher and broader antibacterial activity than the most active EA fraction among the TSE extracts.

#### 2.2.2. Minimum Inhibitory Concentrations (MIC) and Minimum Bactericide Concentration (MBC) Determination

Based on the results from the disk diffusion assay, extracts A and B of SFE, ethanol crude extract, Hex fraction and EA fraction of TSE were selected for MIC and MBC determination. As shown in Table 3, SFE extract B of leaves displayed the best antibacterial activity against Sa with MIC range between 1.0–3.0 mg/mL and MBC range between1.5–4.5 mg/mL. Extract B exhibited antibacterial activities against Pa with MIC range between 0.3–4.0 mg/mL and MBC range between 0.5–4.5 mg/mL, while that against Ab revealed MIC range between 0.1–1.0 mg/mL and MBC range between 0.2–1.5 mg/mL. With the exception of strain Pa3647 which was the least susceptible to the influence of extract B, extract B demonstrated better antibacterial abilities against the Gram negative strains than the Gram positive strains. In contrast to the leaf extracts, the twig extract A revealed greater antibacterial activity against the Gram negative strains than that of the extract B with MIC and MBC ranges between 0.3–0.5 mg/mL and 0.3–0.8 mg/mL, respectively. Among the test strains, the strain Pa3647 exhibited the least susceptibility to influence of the SFE extracts. Among the various extracts of SFE, the leaf extract B had broader antimicrobial spectrum than the twig extract. However, the twig extract B displayed better antimicrobial activity than the leaf extract B against Gram positive strains. The results indicated that different strains of bacteria are susceptible to different degrees of influence from the sample extracts [31].

As shown in Table 3, results for the TSE ethanol crude extracts of leaves and twigs of *I. verum* demonstrated more significant antibacterial effects against Sa with the MIC and MBC ranges of 4.0–5.0 and 5.0–6.0, respectively. Among the partition fractions, the EA fraction revealed higher antibacterial activity than that of the Hex fraction against the Gram negative strains with MIC and MBC ranges of 0.8–3.5 mg/mL and 1.5–4.8 mg/mL, respectively. Among the various extracts of TSE, the ethanol crude extract displayed a higher antibacterial activity against Gram positive strains. However, the partition fractions demonstrated more significant antibacterial against the Gram negative strains with the exception of strain Pa3647. The results indicate that the lower polarity components in SFE extracts provide better antimicrobial activities against the Gram negative strains. Similar results were obtained from the Hex and EA fractions; however, the higher polarity substances in the ethanol extracts played a more significant role in the antibacterial activity against the Gram positive strains. Therefore, different extraction method performed on leaves and twigs of *I. verum* may enable derivation of different activated compounds against distinct pathogens.

According to our previous study [27], the main compounds found within fruits of *I. verum* are anethole, anisyl aldehyde, anisyl acetone and anisyl alcohol. Thus, in this study, antibacterial activity tests were conducted on the main compounds, which are referred to as the standard substances. As shown in Table 4, anethole displayed significant antibacterial activity against Sa with MIC less than 0.1 mg/mL and MBC 0.1 mg/mL. However, anethole did not demonstrate significant antibacterial activity against the Pa and Ab strains. Anisyl aldehyde, anisyl acetone and anisyl alcohol provides a broader antibacterial spectrum against the test strains with MIC and MBC ranges of 1.5–5.0 mg/mL and 2.5–6.5 mg/mL, respectively.

Among the sample extracts, the SFE extract B from leaves showed significant antibacterial activity against Sa2875, Pa4016 and Ab3327 with MIC of 3.0 mg/mL, 0.3 and 0.1 mg/mL and MBC of 4.0 mg/mL, 0.5 mg/mL and 0.2 mg/mL, respectively. The ethanol crude extracts only demonstrated antibacterial effects against Sa with the ranges of MIC and MBC between 4.0–5.0 mg/mL and 5.0–6.0 mg/mL, respectively. The partition fractions (Hex and EA) did not exhibit greater antimicrobial activity than the crude extract against the Sa strains. All of the TSE extracts displayed lower antimicrobial activity than the SFE extracts against all the test strains. Among the standard substances, anisyl aldehyde showed slightly higher antimicrobial activity than the SFE extract B of leaves against strain Sa2875 with MIC and MBC of 2.0 mg/mL and 3.0 mg/mL, respectively. The other standard substances all exhibited lower antimicrobial activity than the SFE extract B of leaves against the test strains. Based on the results obtained, it was revealed that SFE extracts of leaves of *I. verum* displayed excellent antibacterial effects against the test strains. The TSE extracts of EA fraction exerted higher antimicrobial effects than anisyl aldehyde, anisyl acetone and anisyl alcohol against Ab3327. In general, the SFE extract B exhibited a higher antimicrobial activity compared to other sample extracts and standard substances. Hence, the SFE extracts were further subjected to phytochemical component analysis.

Through comparison of our results with the published articles on the antimicrobial effect, we have found that although many reports included similar results to our findings [14,18,32], there were some studies that revealed results contrary to our findings [29,33]. This may be due to differences in bioassays selected, the microorganisms used to carry out the test (microbial strains, inoculum preparation techniques, inoculum size, growth medium, incubation conditions and endpoints determination), and the degree of solubility of each test extract obtained from different extraction methods [34]. In this study, aside from the disk-diffusion and broth dilution methods used, the time-killing test and SEM observation were also employed to analyze the antimicrobial effect of extracts. The use of multiple procedures provide information on the nature of the inhibitory effect, such as ones relating to bactericidal, bacteriostatic, time-dependent, concentration-dependent and cell damaging to the tested microorganisms.

#### 2.2.3. Time-Killing Curve Analysis

**Figure 1:**
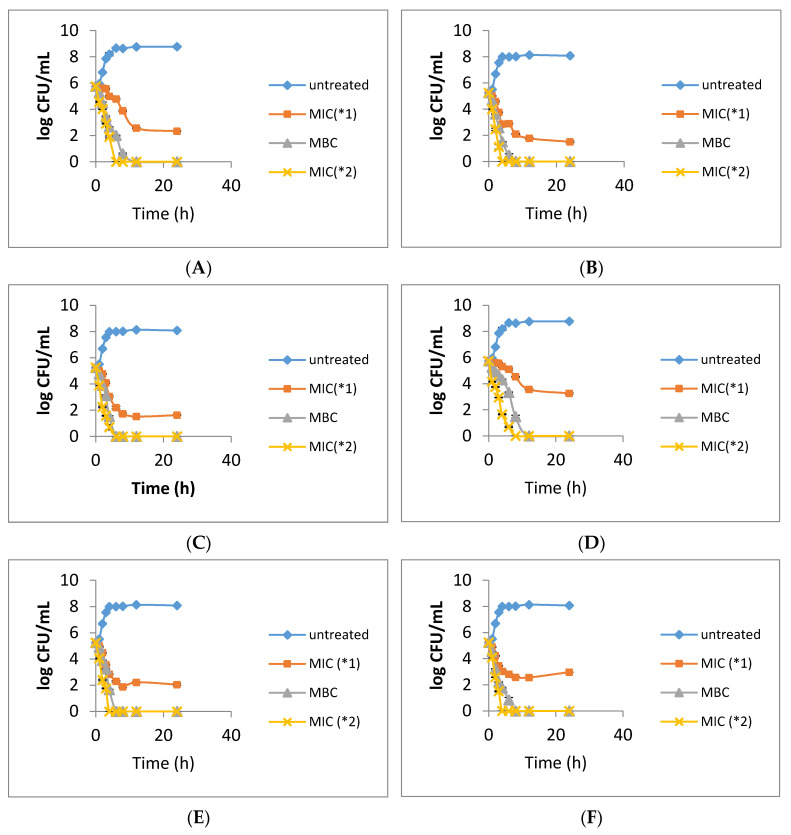

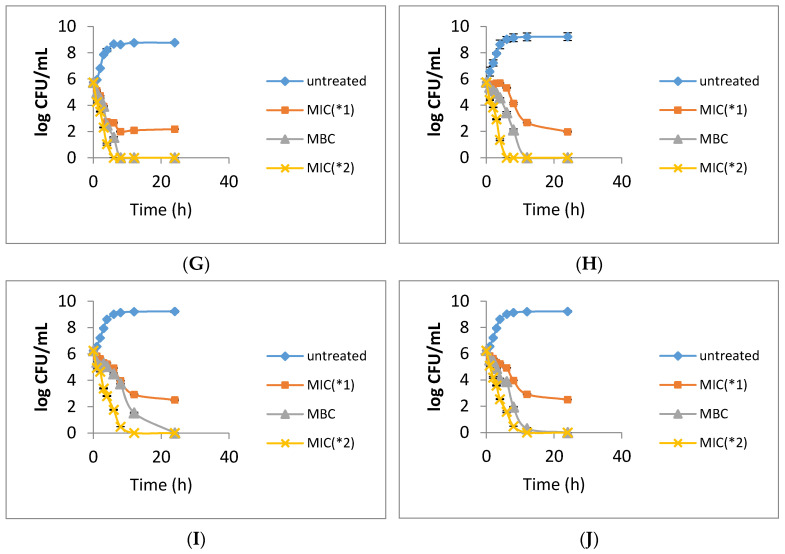
Time–killing curve analysis. (**A**): Ab3394 with leaf SFE extract A; (**B**): Ab3394 with leaf SFE extract B; (**C**): Pa4016 with leaf SFE extract A; (**D**): Pa4016 with leaf SFE extract B; (**E**): Ab3394 with twig SFE extract A; (**F**): Ab3394 with twig SFE extract B; (**G**): Pa4016 with twig SFE extract; (**H**): Pa4016 with twig SFE extract B; (**I**): Sa2875 with leaf ethanol crude extrac; (**J**): Sa2875 with twig ethanol crude extract.

Results from the antibacterial activity tests revealed that the SFE extracts and ethanol crude extracts obtained from leaves and twigs of *I. verum* exhibited significant antibacterial effects against different strains. To prove that the extracts have bactericidal abilities that can last for 24 h, we conducted a time-kill curve analysis. The various sample extracts from leaves and twigs of *I. verum* and the strains Ab, Pa and Sa were selected for the analysis. As shown in Figure 1, the SFE extracts A and B of leaves inhibit the growth of test strain within 6–8 h, while the effect lasted for 24 h under double dose of MIC (Figure 1A–D). Similarly, the extracts A and B of twigs inhibit the growth of test strains within 6–12 h, while the effect lasted for 24 h under double dose of MIC (Figure 1E,H). The ethanol crude extracts of leaves and twigs inhibited the growth of test strain within 8–12 h, while the effect lasted for 24 h under double dose of MIC (Figure 1J). The results revealed that the extracts of leaves and twigs of *I. verum* exhibit bactericide abilities that may enable it to be developed as natural antibiotics against clinical antibiotic resistant pathogens.

### 2.3. Phytochemical Composition Analysis

Based on results from the antimicrobial activity assay, the various SFE extracts of *I. verum* were subjected to GC-MS analysis for chemical component analysis. As shown in Table 5, anethole and anisyl aldehyde were identified as the main active compounds of leaf SFE extracts A and B. However, only anethole was identified in the leaf extracts C and C_Hex_. On the other hand, anisyl acetone was only found in the extract C_EA_. In terms of the twig extracts, anethole, anisyl aldehyde, and anisyl alcohol were identified as the main active compounds in extract A while anethole and anisyl alcohol were identified in extract B. Similar to the leaf extracts, only anethole was identified in extracts C and C_Hex_. With respect to the antibacterial activity and identity of the main components, the results indicated that leaf A and B extracts exhibited the best antibacterial activity, which was correlated to the presence of anethole and anisyl aldehyde. The twig A and B extracts exhibited better antibacterial activities that may be contributed by the presence of anethole, anisyl aldehyde and anisyl alcohol. Based on analysis of the phytochemical components and data of the antimicrobial activities of the standard substances, the results indicate that the presence of multiple components (anisyl aldehyde, anisyl alcohol) within the herbal extract may lead to greater antimicrobial effects.

Similar to our results, previous studies revealed that *trans*-anethole is the most abundant component in the fruit essential oil of *I. verum* as it possessed the highest proportion of 85% among the extract compositions [14,35,36]. However, Zhang et al. reported that *trans*-anethole is only found in the fruit of *I. verum* and not in the leaves [8]. The discrepancy between our results and Zhang’s study may be due to the differences in regard to the test plant materials. The tested plant materials used in our study differed from that of Zhang’s study in many areas, including different geographical environments, growth seasons and physiological age, and the methods of extraction [12]. Based on the results from our investigations, leaf and twig as well as fruit SFE extracts were found to contain *trans*-anethole; however, the fruit SFE extract did not contain the other components, anisyl aldehyde and anisyl alcohol, which were found in the leaf and twig extracts, respectively. Many literatures suggested that *trans*-anethole provided antimicrobial, antifungal, antioxidant and insecticidal activity [12,25,37]. Based on the antibacterial activity assay of standard substances (Table 4), *trans*-anethole exhibited antibacterial activity against Ab but not for Sa and Pa. Therefore, it was concluded that the broader antibacterial spectrum observed from leaf and twig SFE extracts (Table 3), which indicate substantial antibacterial activity against Sa and Pa (Table 4), may be due to the presence of the minor components anisyl aldehyde and alcohol.

### 2.4. Cytotoxicity Assay

**Figure 2:**
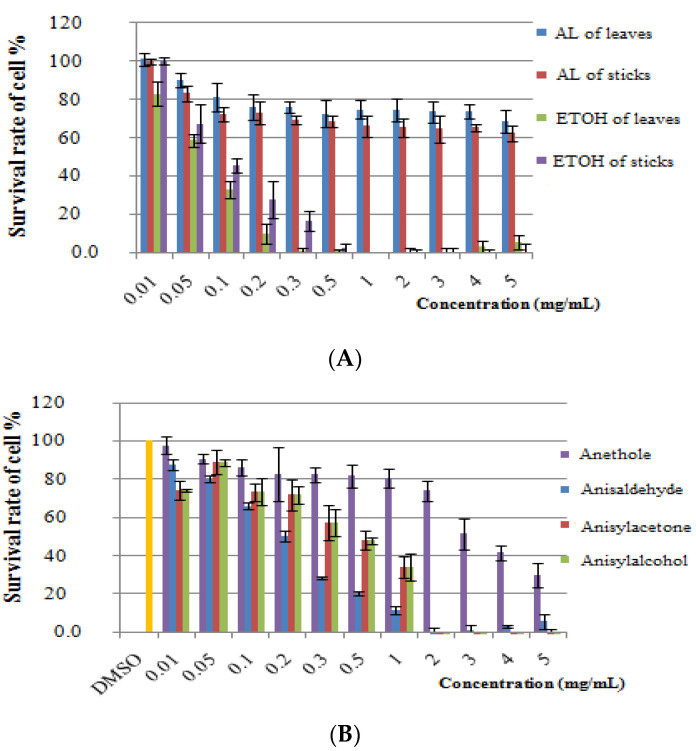
Cytotoxicity assay. (**A**): the various extracts; (**B**): the standard substances.

To determine whether the extracts of leaves and twigs of *I. verum* exert cytotoxic effects on human cell, the 3-(4,5-dimethyl-2-thiazolyl)-2,5-diphenyl-2*H*-tetrazolium bromide (MTT) assay were conducted on the sample constructs. Among the various sample extracts (Figure 2A), the SFE extracts displayed lower toxicity than the ethanol extracts with a concentration of 5 mg/mL as the survival rate of cell was above 60%. The leaf SFE extracts displayed lower toxicity than the twig SFE extracts with a concentration of 0.1 mg/mL as the survival rate of cell was above 80%. The standard substance, anethole, displayed lower toxicity than the others standard substances with the concentration of 1.0 mg/mL, as the survival rate was above 80% (Figure 2B). Nonetheless, all the standard substances exhibited higher toxicity than the SFE extracts. Therefore, the results indicate that the SFE extracts from leaves and twigs of *I. verum* exhibit minimal toxicity toward the test cells.

### 2.5. Scanning Electron Microscopy (SEM) Observation

**Figure 3:**
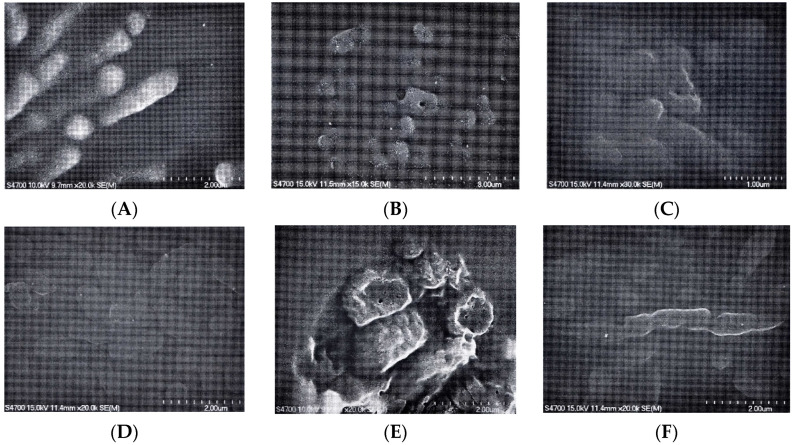

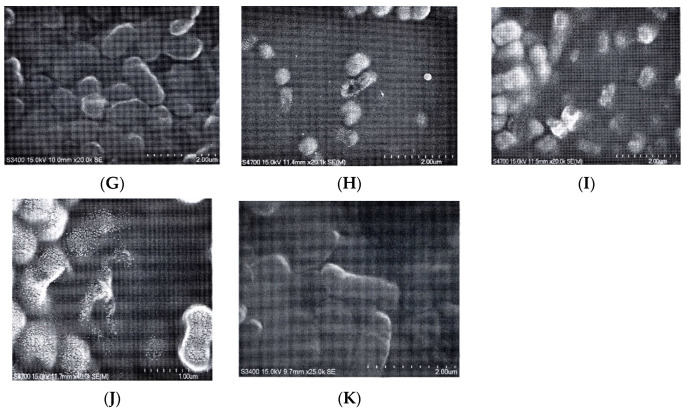
Scanning electron microscopy (SEM) observation. (**A**): Ab3327 without extract; (**B**): Ab3327 with leaf SFE extract B; (**C**): Ab3327 with Anethole; (**D**): Ab3327 with Anisyl aldehyde; €: Ab3327 with Anisyl acetone; (**F**): Ab3327 with Anisyl alcohol; (**G**): Pa4016 without extract; (**H**): Pa4016 with leaf SFE extract B; (**I**): Pa4016 with Anisyl aldehyde; (**J**): Pa4016 with Anisyl acetone; (**K**): Pa4016 with Anisyl alcohol.

To examine the antibacterial mechanism of the test extracts against strains Ab3327 and Pa4016 under half dose of the leaf SFE extract B and the standard substances (anethole, anisyl aldehyde, anisyl acetone and anisyl alcohol) were subjected to SEM observation. As shown in Figure 3, the cells cultivated with the extracts or standard substances manifested conspicuous holes with rough and dehydrated appearances, indicating that the test agents disrupted the membrane integrity of the cells. Similar findings were also reported by many previous studies, especially for antibacterial compounds that are low molecular weight organic agents [38].

Although the antibacterial mechanism of herbal extracts has yet to be discovered, many inferences were made in previous studies. One of the potential mechanisms relates to the critical role of phenolic substances in altering the permeability of the cell membrane. The effects of phenolic substances may lead to irreversible damage in the cytoplasmic membrane and coagulation of the cell contents as the substances engage in hydrogen-binding with intracellular enzymes, triggering loss of enzymatic functions [39,40,41,42,43].

## 3. Materials and Methods

### 3.1. Materials

Leaves and twigs were collected from *I. verum* trees locally grown in Guangxi and Guangdong, China. These herbal materials were authorized by Kaohsiung Medicine University, Taiwan. A total of nine test strains, including *Staphylococcus aureus* (Sa), *Pseudomonas aeruginosa* (Pa) and *Acinetobacter baumannii* (Ab), were isolated from the patients’ blood or sputum from a hospital in Chiayi, Taiwan. The cell, L929 fibroblast, was provided by the Department of Biomedical Engineering in I-Shou University.

### 3.2. Extracts Preparation

The SFE sample extracts were prepared by applying the method described in our previous study [27] with minor modifications. The Chinese medicinal herbs were ground for a short period of time before being placed into a 5 L extraction tank with the pressure being gradually increased up to 276 bar at 50 °C. The extracts were collected during three stages: (a) 30 min after the tanks started to step up when the pressure was below 276 bar; the collected extract was referred as A. (b) when the pressure had reached 276 bar; the collected extract was referred as B. (c) after extractions were completed, the separation tanks were washed with ethanol; the collected extract was referred as C. To prevent extraction of the intermingled compounds of the separation tank, part of extract C was further partitioned using hexane and ethyl acetate, which were referred as C_Hex_ and C_EA_, respectively.

For the traditional solvent extraction (TSE), the ground herbs were extracted by 95% ethanol (1 g:5 mL) at 37 °C, 200 rpm shaker for 24 h. The extraction was repeated three times and the resulting extracts were put through suction filtration at 40 °C. The ethanol crude extracts were then suspended in water and partitioned with hexane and ethyl acetate. The extracts obtained from TSE were referred as ethanol crude extract, Hex fraction, EA fraction and aqueous fraction, respectively.

### 3.3. Antibacterial Activity Test

#### 3.3.1. Disk Diffusion Test

According to the standard protocol described by the National Committee of Clinical Laboratory Standards (NCCLS) [44], nine clinical antibiotic resistant isolates, including Sa2875, Sa2803, Sa0752, Pa4016, Pa4285, Pa3647, Ab3327, Ab2375 and Ab3394, were used in the disk diffusion test. The dried herbal extracts were dissolved in dimethyl sulfoxide (DMSO, 0.1 g/mL) for a brief period of time. Subsequently, 30 µL of the extract solution were added on disks (6 mm in diameter). The impregnated disks were then placed on cation-adjusted Mueller Hinton agar plates, which were inoculated with test organisms (50 µL, OD_600_ = 0.3). DMSO was used as the negative control while tetracycline (TC, 15 mg/mL) was used as the positive control. The plates were incubated for 16 h at 37 °C before measurements of the disk inhibition zone (DIZ) were taken to determine the antibacterial activity of the herbal extracts. The extracts that exhibited antibacterial activity were then subjected to the minimum inhibitory concentration (MIC) and minimum bactericidal concentration (MBC) determination.

#### 3.3.2. Minimum Inhibitory Concentration (MIC) and Minimum Bactericidal Concentration (MBC) Determination

The MIC and MBC determination was conducted by the broth dilution method according to the process of NCCLS [44]. Different concentration of herbal extracts were added into 5 mL of cation-adjusted Mueller-Hinton broth for a brief period of time. DMSO was used as the negative control. Subsequently, 50 µL diluted bacterial culture, which had been cultivated for 12–16 h to 10^7^ CFU/mL, was added into the broth with the sample extract. The bacterial culture was then incubated at 37 °C, 200 rpm, for 16 h. The lowest concentration which inhibited 99% of the growth of the respective microorganisms was taken as the MIC. The lowest concentration which inhibited 100% of the growth of the respective microorganisms was taken as the MBC. All tests were carried out in triplicate.

#### 3.3.3. Time-Killing Curve

By administrating the method described in our previous study [27] with some modification, 50 µL diluted bacterial liquid (10^7^ CFU/mL) was inoculated to 5 mL of the cation-adjusted Mueller-Hinton broth with different concentration of extracts, which includes 1x MIC, 2x MIC and 1x MBC, followed by incubation at 37 °C, 200 rpm. 100 µL of the bacterial culture was taken at 0, 1, 2, 3, 4, 6, 8, 12 and 24 h, before measurements of the total viable bacteria number were taken. Cell culture without extracts served as the control.

### 3.4. Phytochemical Composition Analysis

Based on our previous study [45] with some modification, the extracts were dissolved in methanol followed by GC-MS analysis on an Agilent system (Agilent Technologies, Inc., Santa Clara, CA, USA), which consisted of a model 6890N gas chromatographer and a model 5975A mass selective detector (MSD, electron energy, 70 eV) using a DB-5 column (60 m × 250 μm × 0.25 μm). The carrier gas was helium (99.99%) with a flow rate of 0.8 mL/min. The injector and detector temperatures were set at 270 °C and 270 °C respectively. Spectra were obtained over a scan range of 50 to 550 amu at 2 scans/s. The GC program was designed as follows: the initial temperature was 80 °C and held for 5 min, then increased by 5 °C/min to 180 °C and held for 5 min, then raised by 20 °C/min to 270 °C and held for 5 min. For comparative studies, experiments were also conducted in a similar manner using (*E*)-anethole, anisyl aldehyde, anisyl acetone, and anisyl alcohol as standards.

### 3.5. Cytotoxicity Assay

Cytotoxicity of the sample extracts was evaluated by conducting the 3-(4,5-dimethyl-2- thiazolyl)-2,5-diphenyl-2*H*-tetrazolium bromide (MTT) assay [46]. Briefly, mouse fibroblast (L929) cells (1 × 10^4^ cells/well) were cultured in Dulbecco’s modified Eagle’s medium (Hyclone, Logan, UT, USA) containing 10% fetal bovine serum and penicillin-streptomycin, after cultivation in an incubator (37 °C, 5% CO_2_) for 24 h in a 96-well plate. Subsequently, the original medium was removed and applied with 100 µL of the new medium and 2 µL of the different concentrations of the test substance, followed by cultivation for 24 h. Finally, 100 µL of MTT (1mg/mL) was added and incubated for 1.5 h while dimethyl sulfoxide was used to dissolve the formazan crystals. The absorbance at 570 nm was measured using a Digiscan microplate reader (Assys Hitech, Kornenburg, Austria). The well without cells was used as the background blank as its absorbance was subtracted from that of each test sample.

### 3.6. Scanning Electron Microscope (SEM) Observation

The SEM observation was examined by the method described in our previous study [2]. Briefly, the bacterial suspension was inoculated in 5 mL cation-adjusted Mueller-Hinton broth containing the test substance (the extracts, (*E*)-anethole, anisyl aldehyde, anisyl acetone, and anisyl alcohol). The bacterial culture was incubated at 37 °C for 12 h; the cells were then harvested by centrifugation at 8000 rpm and prefixed with 5% glutaraldehyde (Sigma, St. Louis, MO, USA) in 0.1 M cacodylate buffer (pH 7.2) at 4 °C for 1.5 h. After being washed with the buffer, specimens were post fixed for 1 h with 1% osmium tetroxide in 0.1 M cacodylate buffer (pH 7.4) at 4 °C. The samples were dehydrated through a series of 30%, 50%, 70%, 90%, 100% ethanol and dried at room temperature. Subsequently, the dried samples were treated by gold-coverings with catholic spraying. The samples were examined using a S-2700 Scanning Electron Microscope (HITACHI, Tokyo, Japan).

### 3.7. Statistical Analysis

The data was analyzed using the analysis of variance (ANOVA) with Tukey HSD test. A *p*-value of less than 0.05 was considered statistically significance. The experimental results were expressed as mean ± standard deviation (SD) of three replicates.

## 4. Conclusions

In recent years, there has been a shift in research interest to the recycling of resources. In this study, the leaf and twig constituents of *Illicium verum* were obtained from the waste of trees pruned after each harvest. Potential of the trimmings possessing active biological properties would enable greatly enhance the value of *I. verum* cultivation. Numerous literature reports have demonstrated that *I. verum* fruits exert significant antimicrobial activity against various different types of microorganism while the main component, trans-anithole, plays a critical role in various bioactivities observed from the fruit [10,11,12,13]. In this study, experimental results from utilizing the SFE extracts obtained from leaves and twigs of *I. verum* indicated that the extracts are capable of inducing significant antimicrobial activities against the clinical drug resistant pathogens. Hence, it was concluded that the broader antibacterial spectrum may be attributed to the presence of multiple active components. Nonetheless, further research on the mechanism of antibacterial action may enable us to obtain additional insights on traditional Chinese medicine theory and the relation between traditional uses and modern pharmacology of this herbal medicine. In addition, future studies must also address the possible synergistic effect among multiple active compounds within the extracts, such as ones relating to the combination ratio of *trans*-anethole, anisyl aldehyde, anisyl acetone and anisyl alcohol. The present study also demonstrated that the active extracts exhibited very little toxicity to the test cells. To the best of our knowledge, not much progress has been made in regard to the antimicrobial activity against clinical antibiotic resistant isolates of the *I. verum* leaves and twigs [26]. This is the first report on the comparison of phytochemical contents and antimicrobial activities against the clinical antibiotic resistant pathogens from the various extracts of *I. verum* leaves and twigs. The active compounds may serve as potential candidates for future in-depth studies on synergism from multiple compounds and development of commercial natural antimicrobial agents.

## Figures and Tables

**Table 1:** Extraction yields (%).

Extracts *	Supercritical CO_2_ Extraction (SFE)					Traditional Solvent Extraction (TSE)			
	A	B	C	C_Hex_	C_EA_	Ethanol Crude Extract	Hex Fraction	EA Fraction	Aqueous Fraction
leaves	0.59	0.26	0.14	0.11	0.16	14.27	2.87	1.22	0.41
twigs	0.96	0.28	0.17	0.09	0.17	16.1	3.37	1.42	0.62

* A: The extracts collected from the first 30 min; B: The extracts collected from the second 30 min; C: The extracts collected from the separation tanks washed ethanol after extractions; C_Hex_: The hexane fraction from the partition extraction of extract C; C_EA_: The ethyl acetate fraction from the partition extraction of extract C; Hex fraction: The hexane fraction from the partition extraction of ethanol crude extract; EA fraction: The ethyl acetate fraction from the partition extraction of ethanol crude extract; Aqueous fraction: The aqueous fraction from the partition extraction of ethanol crude extract.

**Table 2:** Disk Inhibitory zone (mm) of the various extracts.

Test Strains	Supercritical CO_2_ Extraction										Tetracycline	Traditional Solvent Extraction							
	Leaves *					Twigs *						Leaves *				Twigs			
	A	B	C	C_Hex_	C_EA_	A	B	C	C_Hex_	C_EA_		Ethanol Crude Extract	Hex Fraction	EA Fraction	Aqueous Fraction	Ethanol Crude Extract	Hex Fraction	EA Fraction	Aqueous Fraction
Sa2875	11.0 ± 0.0	16.5 ± 1.4	18.5 ± 0.7	-	11.5 ± 0.7	-	18.5 ± 0.7	9.5 ± 0.7	9.5 ± 0.7	-	14.5 ± 0.7	12.0 ± 1.4	9.0 ± 0.0	11.5 ± 0.7	-	15.0 ± 1.4	11.0 ± 0.0	15.5 ± 0.7	-
Sa2803	10.0 ± 0.0	14.0 ± 0.0	10.0 ± 0.0	-	9.0 ± 0.0	-	10.0 ± 0.0	-	-	-	25.5 ± 2.1	13.5 ± 0.7	11.0 ± 0.0	12.5 ± 0.7	-	17.5 ± 0.7	9.5 ± 0.7	10.0 ± 0.0	-
Sa0752	9.0 ± 0.0	14.0 ± 0.0	11.5 ± 0.7	-	10.0 ± 0.0	-	10.0 ± 0.0	9.0 ± 0.0	-	-	26.0 ± 1.4	13.0 ± 0.0	10.0 ± 0.0	10.5 ± 0.0	-	19.0 ± 1.4	9.0 ± 0.0	9.0 ± 0.0	-
Pa4016	16.5 ± 0.7	16.5 ± 1.4	13.5 ± 0.7	-	9.5 ± 0.7	14.5 ± 0.7	15.5 ± 0.7	12.0 ± 0.0	10.5 ± 0.7	10.0 ± 0.0	15.0 ± 0.0	10.0 ± 0.0	10.0 ± 0.0	12.5 ± 0.7	-	9.5 ± 0.7	12.0 ± 0.0	16.5 ± 0.7	-
Pa4285	15.0 ± 0.0	17.0 ± 1.4	12.0 ± 0.0	-	9.0 ± 0.0	17.5 ± 0.7	18.5 ± 0.7	15.0 ± 0.0	9.0 ± 0.0	9.5 ± 0.7	17.5 ± 0.7	9.0 ± 0.7	12.0 ± 0.0	12.5 ± 0.7	-	10.0 ± 0.7	13.0 ± 0.0	15.0 ± 1.4	-
Pa3647	12.5 ± 0.7	14.0 ± 0.0	10.5 ± 0.7	-	-	9.0 ± 0.0	10.0 ± 0.0	10.0 ± 0.0	-	-	24.0 ± 0.0	-	9.5 ± 0.0	10.0 ± 0.0	-	-	11.5 ± 0.7	13.0 ± 0.0	-
Ab3327	22.0 ± 1.4	20.5 ± 0.7	15.5 ± 0.7	-	11 ± 0.0	22.0 ± 0.0	22.0 ± 0.0	14.5 ± 0.7	11.0 ± 0.0	12.0 ± 0.0	28.5 ± 0.7	11.5 ± 0.7	17.0 ± 1.4	15.0 ± 1.4	10.5 ± 0.7	10.0 ± 0.0	14.0 ± 0.0	22.5 ± 2.1	13.0 ± 0.0
Ab2375	19.0 ± 1.4	16.5 ± 0.7	10.5 ± 0.7	-	-	15.0 ± 0.0	18.0 ± 1.4	14.5 ± 3.5	-	9.0 ± 0.0	-	9.0 ± 0.0	14.5 ± 0.7	16.0 ± 0.0	-	10.5 ± 0.7	15.0 ± 1.4	19.5 ± 0.7	9.0 ± 0.0
Ab3394	14.0 ± 2.1	18.0 ± 1.4	11.5 ± 0.7	-	9.5 ± 0.7	13.0 ± 0.0	19.5 ± 0.7	16.5 ± 2.1	-	9.5 ± 0.7	24.0 ± 1.4	9.0 ± 0.0	12.5 ± 0.7	15.5 ± 0.7	-	9.5 ± 0.7	12.0 ± 0.0	14.0 ± 0.0	-

-: no inhibitory zone. *: A: The extracts collected from the first 30 min; B: The extracts collected from the second 30 min; C: The extracts collected from the separation tanks washed ethanol after extractions; C_Hex_: The hexane fraction from the partition extraction of extract C; C_EA_: The ethyl acetate fraction from the partition extraction of extract C.

**Table 3:** Minimum inhibitory concentrations (MIC, mg/mL) and minimum bactericide concentration (MBC, mg/mL) of the various extracts of *Illicium verum*.

Test Strain	Supercritical CO_2_ Extraction																Traditional Solvent Extraction											
	Leaves *								Twigs *								Leaves						Twigs					
	A		B		C		C_EA_		A		B		C		C_EA_		Ethanol Crude Extract		Hex Fraction		EA Fraction		Ethanol Crude Extract		Hex Fraction		EA Fraction	
	MIC	MBC	MIC	MBC	MIC	MBC	MIC	MBC	MIC	MBC	MIC	MBC	MIC	MBC	MIC	MBC	MIC	MBC	MIC	MBC	MIC	MBC	MIC	MBC	MIC	MBC	MIC	MBC
Sa2875	>5	>5	3.0 ± 0.0	4.0 ± 0.0	>5	>5	>5	>5	>5	>5	>5	>5	>5	>5	>5	>5	5.0 ± 0.0	6.0 ± 0.0	>5	>6	5.0 ± 0.0	6.0 ± 0.0	4.0 ± 0.0	5.5 ± 0.0	5.0 ± 0.0	6.0 ± 0.0	4.5 ± 0.0	5.0 ± 0.0
Sa2803	>5	>5	1.0 ± 0.0	1.5 ± 0.0	5.0 ± 0.0	>5	>5	>5	>5	>5	5.0 ± 0.0	>5	>5	>5	>5	>5	4.8 ± 0.0	6.0 ± 0.0	>5	>6	5.0 ± 0.0	5.8 ± 0.0	5.0 ± 0.0	5.8 ± 0.0	>5	>6	>5	>6
Sa0752	>5	>5	3.0 ± 0.0	4.5 ± 0.0	5.0 ± 0.0	>5	>5	>5	>5	>5	5.0 ± 0.0	>5	5.0 ± 0.0	>5	>5	>5	5.0 ± 0.0	5.5 ± 0.0	>5	>6	>5	>6	4.0 ± 0.0	5.0 ± 0.0	>5	>6	>5	>6
Pa4016	1.0 ± 0.0	1.5 ± 0.0	0.3 ± 0.0	0.5 ± 0.0	>5	>5	>5	>5	0.3 ± 0.0	0.5 ± 0.0	0.5 ± 0.0	0.8 ± 0.0	>5	>5	>5	>5	>5	>6	>5	>6	4.0 ± 0.0	5.0 ± 0.0	>5	>6	5.0 ± 0.0	6.0 ± 0.0	2.0 ± 0.0	2.8 ± 0.0
Pa4285	1.0 ± 0.0	1.5 ± 0.0	1.0 ± 0.0	1.5 ± 0.0	>5	>5	>5	>5	0.3 ± 0.0	0.5 ± 0.0	0.3 ± 0.0	0.5 ± 0.0	>5	>5	>5	>5	>5	>6	4.8 ± 0.0	5.5 ± 0.0	4.5 ± 0.0	5.5 ± 0.0	>5	>6	4.0 ± 0.0	4.5 ± 0.0	1.5 ± 0.0	2.5 ± 0.0
Pa3647	4.0 ± 0.0	5.0 ± 0.0	4.0 ± 0.0	4.5 ± 0.0	>5	>5	>5	>5	0.5 ± 0.0	0.8 ± 0.0	>5	>5	>5	>5	>5	>5	>5	>6	>5	>6	>5	>6	>5	>6	5.0 ± 0.0	5.8 ± 0.0	3.5 ± 0.0	4.8 ± 0.0
Ab3327	3.0 ± 0.0	3.5 ± 0.0	0.1 ± 0.0	0.2 ± 0.0	>5	>5	>5	>5	0.3 ± 0.0	0.5 ± 0.0	0.1 ± 0.0	0.2 ± 0.0	>5	>5	>5	>5	>5	>6	3.0 ± 0.0	3.8 ± 0.0	1.3 ± 0.0	2.0 ± 0.0	>5	>6	3.5 ± 0.0	4.3 ± 0.0	0.8 ± 0.0	1.5 ± 0.0
Ab2375	0.8 ± 0.0	1.0 ± 0.0	1.0 ± 0.0	1.5 ± 0.0	>5	>5	>5	>5	0.3 ± 0.0	0.5 ± 0.0	0.3 ± 0.0	0.5 ± 0.0	>5	>5	>5	>5	>5	>6	4.0 ± 0.0	5.0 ± 0.0	1.5 ± 0.0	2.3 ± 0.0	>5	>6	2.0 ± 0.0	2.5 ± 0.0	1.0 ± 0.0	1.5 ± 0.0
Ab3394	0.3 ± 0.0	0.5 ± 0.0	1.0 ± 0.0	1.5 ± 0.0	>5	>5	>5	>5	0.3 ± 0.0	0.5 ± 0.0	0.3 ± 0.0	0.5 ± 0.0	>5	>5	>5	>5	>5	>6	5.0 ± 0.0	6.3 ± 0.0	2.3 ± 0.0	3.5 ± 0.0	>5	>6	5.0 ± 0.0	6.0 ± 0.0	3.0 ± 0.0	4.0 ± 0.0

*: A: The extracts collected from the first 30 min; B: The extracts collected from the second 30 min; C: The extracts collected from the separation tanks washed ethanol after extractions; CEA: The ethyl acetate fraction from the partition extraction of extract C.

**Table 4:** Disk Inhibitory zone (DIZ, mm), minimum inhibitory concentrations (MIC, mg/mL) and minimum bactericide concentration (MBC, mg/mL) of the standard substances.

Test Strains	Anethole			Anisyl Aldehyde			Anisyl Acetone			Anisyl Alcohol		
	DIZ	MIC	MBC	DIZ	MIC	MBC	DIZ	MIC	MBC	DIZ	MIC	MBC
Sa2875	-	>5	>5	20.0 ± 1.4	2.0 ± 0.0	3.0 ± 0.0	15.0 ± 1.4	5.0 ± 0.0	6.5 ± 0.0	19.0 ± 0.0	3.0 ± 0.0	5.0 ± 0.0
Pa4016	10.5 ± 0.7	>5	>5	22.0 ± 2.8	1.5 ± 0.0	2.5 ± 0.0	19.0 ± 1.4	2.0 ± 0.0	3.0 ± 0.0	20.5 ± 0.7	2.0 ± 0.0	3.5 ± 0.0
Ab3327	10.5 ± 0.7	<0.1	0.1 ± 0.0	21.0 ± 0.0	2.0 ± 0.0	3.0 ± 0.0	16.0 ± 1.4	2.0 ± 0.0	3.5 ± 0.0	20.0 ± 0.0	2.0 ± 0.0	3.0 ± 0.0

**Table 5:** Main active compounds of the extracts of supercritical CO_2_ extraction by GC-MS analysis.

Extracts *		Anethole	Anisyl Aldehyde	Anisyl Acetone	Anisyl Alcohol	Unknown
Leaves	A	95.15%	1.82%	-	-	3.03%
	B	88.67%	7.38%	-	-	3.95%
	C	80.42%	-	-	-	19.58%
	C_Hex_	100%	-	-	-	-
	C_EA_	-	-	100%	-	-
Twigs	A	97.30%	0.21%	-	0.57%	1.92%
	B	97.42%	-	-	1.44%	1.14%
	C	99.98%	-	-	-	0.02%
	C_Hex_	75.51%	-	-	-	24.49%
	C_EA_	83.45%	-	-	-	16.55%

-: Compound does not exit. *: A: The extracts collected from the first 30 min; B: The extracts collected from the second 30 min; C: The extracts collected from the separation tanks washed ethanol after extractions; C_Hex_: The hexane fraction from the partition extraction of extract C; C_EA_: The ethyl acetate fraction from the partition extraction of extract C.

## Data Availability

Not applicable.

## Sample Availability

Not available.

## References

[1] Su P.W., Yang C.H., Yang J.F., Su P.Y., Chuang L.Y. (2015). Antibacterial activities and antibacterial mechanism of *Polygonum cuspidatum* extracts against nosocomial drug-resistant pathogens. Molecules..

[2] Valle D.L., Cabrera E.C., Puzon J.J., Rivera W.L. (2016). Antimicrobial Activities of Methanol, Ethanol and Supercritical CO_2_ Extracts of Philippine Piper betle L. on Clinical Isolates of Gram Positive and Gram Negative Bacteria with Transferable Multiple Drug Resistance. PLoS ONE.

[3] Santajit S., Indrawattana N. (2016). Mechanisms of antimicrobial resistance in ESKAPE pathogens. Biomed Res Int..

[4] Govindaraj Vaithinathan A., Vanitha A. (2018). WHO global priority pathogens list on antibiotic resistance: An urgent need for action to integrate One Health data. Perspect Public Health..

[5] Yang C.H., Su P.W., Moi S.H., Chuang L.Y. (2019). Biofilm formation in *Acinetobacter baumannii*: Genotype-phenotype correlation. Molecules.

[6] Wang J., Sasse A., Sheridan H. (2019). Traditional Chinese medicine: From aqueous extracts to therapeutic formulae, *Plant Extracts*, Aman Dekebo. IntechOpen.

[7] Akanda M.J., Sarker M.Z., Ferdosh S., Manap M.Y., Ab Rahman N.N., Ab Kadir M.O. (2012). Applications of supercritical fluid extraction (SFE) of palm oil and oil from natural sources. Molecules.

[8] Zhang Q.W., Lin L.G., Ye W.C. (2018). Techniques for extraction and isolation of natural products: A comprehensive review. Chin Med..

[9] Zahid M.S., Awasthi S.P., Hinenoya A., Yamasaki S. (2015). Anethole inhibits growth of recently emerged multidrug resistant toxigenic Vibrio cholerae O1 El Tor variant strains in vitro. J. Vet. Med. Sci..

[10] Wang G.W., Hu W.T., Huang B.K., Qin L.P. (2011). *Illicium verum*: A review on its botany, traditional use, chemistry and pharmacology. J. Ethnopharmacol..

[11] Salem M.A., El-Shiekh R.A., Hashem R.A., Hassan M. (2021). In vivo antibacterial activity of Star Anise (*Illicium verum* Hook.) extract using murine MRSA skin infection model in relation to its metabolite profile. Infect Drug Resist..

[12] Huang Y., Zhao J., Zhou L., Wang J., Gong Y., Chen X., Guo Z., Wang Q., Jiang W. (2010). Antifungal activity of the essential oil of *Illicium verum* fruit and its main component trans-anethole. Molecules.

[13] D’Souza S.P., Chavannavar S.V., Kanchanashri B., Niveditha S.B. (2017). Pharmaceutical perspectives of spices and condiments as alternative antimicrobial remedy. J. Evid. Based Complementary Altern. Med..

[14] Singh G., Maurya S., De Lampasona M.P., Catalan C. (2006). Chemical constituents, microbial investigation and anti-oxidative potential of volatile oil and acetone extract of star anise fruits. J. Sci. Food Agric..

[15] Padmashree A., Roopa N., Semwal A.D., Sharma G.K., Agathian G., Bawa A.S. (2007). Star anise (*Illicium verum*) and black caraway (Carum nigrum) as natural antioxidents. Food Chem..

[16] Chouksey D., Sharma P., Pawar R.S. (2010). Biological activities and chemical constituents of *Illicium verum* hook fruits (Chinese star anise). Der Pharm. Sin..

[17] Ebani V.V., Nardoni S., Bertelloni F., Pistelli L., Mancianti F. (2018). Antimicrobial Activity of Five Essential Oils against Bacteria and Fungi Responsible for Urinary Tract Infections. Molecules.

[18] De M., De A.K., Sen P., Banerjee A.B. (2002). Antimicrobial properties of star anise (*Illicium verum* Hook f). Phytother. Res..

[19] Yazdani D., Rezazadeh S., Amin G., ZainalAbidin M.A., Shahnazi S., Jamalifar H. (2009). Antifungal activity of dried extracts of anise (*Pimpinella anisum* L.) and Star anise (*Illicium verum* hook. f.) against Dermatophyte and Saprophyte fungi. J. Med. Plants.

[20] Fujita K., Tatsumi M., Ogita A., Kubo I., Tanaka T. (2014). Anethole induces apoptotic cell death ac-companied by reactive oxygen species production and DNA fragmentation in *Aspergillus fumigatus* and *Saccharomyces cerevisiae*. FEBS J..

[21] Fujita K., Fujita T., Kubo I. (2007). Anethole, a potential antimicrobial synergist, converts a fungistatic dodecanol to a fungicidal agent. Phytother. Res..

[22] Yi Q., Liu J., Zhang Y., Qiao H., Chen F., Zhang S., Guan W. (2021). Anethole attenuates enterotoxigenic Escherichia coli-induced intestinal barrier disruption and in-testinal inflammation via modification of TLR signaling and intestinal microbiota. Front. Microbiol..

[23] Wei L., Hua R., Li M., Huang Y., Li S., He Y., Shen Z. (2014). Chemical composition and biological activity of star anise *Illicium verum* extracts against maize weevil, *Sitophilus zeamais* adults. J. Insect Sci..

[24] Wieczyńska J., Cavoski I. (2018). Antimicrobial, antioxidant and sensory features of eugenol, carvacrol and trans-anethole in active packaging for organic ready-to-eat iceberg lettuce. Food Chem..

[25] Kwiatkowski P., Grygorcewicz B., Pruss A., Wojciuk B., Dołęgowska B., Gie-drys-Kalemba S., Sienkiewicz M., Wojciechowska-Koszko I. (2019). The Effect of subinhibitory concentrations of trans-anethole on antibacterial and antibiofilm activity of mupirocin against mupirocin-resistant *Staphylococcus aureus* strains. Microb. Drug Resist..

[26] Patra J.K., Das G., Bose S., Banerjee S., Vishnuprasad C.N., Del Pilar Rodriguez-Torres M., Shin H.S. (2020). Star anise (*Illicium verum*): Chemical compounds, antiviral properties, and clinical relevance. Phytother. Res..

[27] Yang J.F., Yang C.H., Chang H.W., Yang C.S., Wang S.M., Hsieh M.C., Chuang L.Y. (2010). Chemical composition and antibacterial activities of *Illicium verum* against antibiotic-resistant pathogens. J. Med. Food..

[28] Rahman M.R., Lou Z., Zhang J., Yu F., Timilsena Y.P., Zhang C., Zhang Y., Bakry A.M. (2017). Star An-ise (*Illicium verum* Hook. f.) as quorum sensing and biofilm formation inhibitor on foodborne bacteria: Study in milk. J. Food Prot..

[29] Li H., Wu X., Li X., Cao X., Li Y., Cao H., Men Y. (2021). Multistage extraction of star anise and black pepper derivatives for antibacterial, antioxidant, and anticancer activity. Front. Chem..

[30] Chemat F., Abert-Vian M., Fabiano-Tixier A.S., Strube J., Uhlenbrock L., Gunjevic V., Cravotto G. (2019). Green extraction of natural products origins, current status, and future challenges. Trend Anal. Chem..

[31] Mostafa A.A., Al-Askar A.A., Almaary K.S., Dawoud T.M., Sholkamy E.N., Bakri M.M. (2018). Antimicrobial activity of some plant extracts against bacterial strains causing food poisoning diseases. Saudi J. Biol. Sci..

[32] Shan B., Cai Y.Z., Brooks J.D., Corke H. (2007). The in vitro antibacterial activity of dietary spice and medicinal herb extracts. Int. J. Food Microbiol..

[33] Benmalek Y., Yahia O.A., Belkebir A., Fardeau M.L. (2013). Anti-microbial and anti-oxidant activities of *Illicium verum*, *Crataegus oxyacantha* ssp monogyna and *Allium cepa* red and white varieties. Bioengineered.

[34] Balouiri M., MSadiki M., Koraichi Ibnsouda S. (2016). Methods for in vitro evaluating antimicrobial activity: A review. J. Pharm. Anal..

[35] Della Porta G., Taddeo R., D’urso E., Reverchon E. (1998). Isolation of clove bud and star anise essential oil by supercritical CO_2_ extraction, *Lebensm*. Wiss. Technol..

[36] Kang G., Mishyna M., Appiah K.S., Yamada M., Takano A., Prokhorov V., Fujii Y. (2019). Screening for plant volatile emissions with allelopathic activity and the identification of L-Fenchone and 1,8-Cineole from star anise (*Illicium verum*) leaves. Plants.

[37] Shahat A.A., Ibrahim A.Y., Hendawy S.F., Omer E.A., Hammouda F.M., Abdel-Rahman F.H., Saleh M.A. (2011). Chemical composition, antimicrobial and antioxidant activities of essential oils from organically cultivated fennel cultivars. Molecules.

[38] Veselov M.S., Sergiev P.V., Osterman I.A., Skvortsov D.A., Golovina A.Y., Andreyanova E.S., Laptev I.G., Pletnev P.I., Evfratov S.A., Marusich E.I. (2015). Common features of antibacterial compounds: An analysis of 104 compounds library. Biomed. Khim..

[39] Bouarab-Chibane L., Forquet V., Lantéri P., Clément Y., Léonard-Akkari L., Oulahal N., Degraeve P., Bordes C. (2019). Antibacterial properties of polyphenols: Characterization and QSAR (Quantitative Structure-Activity Relationship) models. Front. Microbiol..

[40] Delcour A. (2009). Outer membrane per-meability and antibiotic resistance. Biochim. Biophy. Acta.

[41] Nikai-do H. (1988). Bacterial resistance to antibiotics as a function of outer membrane permeability. J. Antimicrob. Ther..

[42] Nikaido H., Vaara M. (1985). Molecular basis of bacterial outer membrane permeability. Microbiol. Rev..

[43] Vaara M. (1993). Antibiotic-supersusceptible mutants of *Escherichia coli* and *Salmonella typhimurium*. Antimicrob. Agents Chemother..

[44] Gressner A.M., Gressner O.A., Gressner A.M., Arndt T. (2019). National Committee for Clinical Laboratory Standards. Lexikon der Medizinischen Laboratoriumsdiagnostik. Springer Reference Medizin.

[45] Yang J.F., Yang C.H., Liang M.T., Gao Z.J., Wu Y.W., Chuang L.Y. (2016). Chemical composition, antioxidant, and antibacterial activity of wood vinegar from *Litchi chinensis*. Molecules.

[46] Vajrabhaya L., Korsuwannawong S. (2018). Cytotoxicity evaluation of a Thai herb using tetrazolium (MTT) and sulforhodamine B (SRB) assays. J. Anal. Sci. Technol..

